# Editorial: 2022 in review: rhinology

**DOI:** 10.3389/falgy.2024.1405836

**Published:** 2024-04-05

**Authors:** Diego M. Conti, Glenis K. Scadding

**Affiliations:** ^1^ The European Forum for Research and Education in Allergy and Airway Diseases Scientific Expert Team Members, Brussels, Belgium; ^2^ Escuela de Doctorado UAM, Centro de Estudios de Posgrado, Universidad Autónoma de Madrid, Madrid, Spain; ^3^ Department of Allergy & Rhinology, Royal National ENT Hospital, London, United Kingdom; ^4^ Division of Immunity and Infection, University College, London, United Kingdom

**Keywords:** rhinology, allergic rhinitis, chronic rhinosinusitis, EUFOREA, AERD, common cold, facial mask, nasal polyp syndrome

**Editorial on the Research Topic** 2022 in review: rhinology

## Introduction

Chronic respiratory diseases (CRD) often begin in early childhood and persist throughout life. They are a major economic burden and have a significant impact on society, causing global health and social inequalities within and between countries (1–3). Asthma, CRS and allergic rhinitis (AR) are the most common non-communicable diseases in children and their prevalence and burden have increased in recent decades to epidemic proportions. All are often underappreciated, misdiagnosed, and ineffectively treated, leading to personal misery and societal losses (4, 5). In this Editorial, we review the key findings of several articles in our Research Topic, addressing the clinical features, mechanisms, and management of CRS and allergies, as well as implications for patient care.

## Allergic rhinitis

Albloushi and Al-Ahmad, reviewed the role of immune mediators, genetic and environmental factors in pathogenesis of allergic rhinitis (AR). Despite well-defined clinical phenotypes of chronic rhinitis, the underlying pathophysiological mechanisms of AR are still not fully understood. This paper provides up-to-date information on the pathogenesis of AR, emphasizing the role of cytokines in adults and investigating the impact of genetic and environmental factors on its pathogenesis.

Oğuz et al., have thoroughly researched the role of face masks in preventing AR symptoms. Their valuable contribution has emphasized aeroallergens as microscopic airborne particles that trigger nasal and ocular symptoms and notes how face masks improve quality of life of AR patients and reduce their symptom severity.

## Chronic rhinosinusitis

Theeling et al., with the support of the patient advisory board of the European Forum for Research and Education in Allergy and Airway Diseases (EUFOREA), have focused on the problems of CRS patients with nasal polyps (CRSwNP), particularly the complex nature of their disease nomenclature. In order to facilitate their medical care and the patient-doctor interaction, they proposed a patient-centred term for CRSwNP: Nasal Polyp Syndrome. This exercise in patient advocacy was very well received and Nasal Polyp Syndrome promises to become a common term.

James et al., entered the world of aroma and set out to determine the impact of CRS on retronasal olfaction (RNO). Based on the current literature, they concluded that patients with CRS appear to have a decrease in RNO, which may be associated with both orthonasal olfactory dysfunction and decreased quality of life. Further studies are needed to better elucidate these relationships and the impact of medical and surgical treatment of CRS on RNO.

## Airway care

Conti et al., set out to answer the question: Is endoscopic sinus surgery sufficient to modify the course of adult aspirin-exacerbated respiratory disease? After a review of the current literature, they concluded that surgery is beneficial in those patients, but its effects are short-lived. Surgery should be considered initially, followed by aspirin desensitization plus aspirin therapy after desensitization, due to the sustained improvement achieved, compared to those receiving endoscopic sinus surgery alone. For those unable or unwilling to undertake aspirin desensitization, or unresponsive to it, biologics can now be considered, where available and affordable.

Eccles, reviewed current knowledge on the common cold, discussing the multiple respiratory viruses that can cause this illness. Factors influencing its incidence are discussed, the mechanism of symptoms is explained in relation to the innate immune response, and the morbidity associated with the common cold and possible vaccines are analyzed.

Hellings et al., presented the EUFOREA Summit 2023 under the theme “inspiring the future of allergy & respiratory care”. The aim was to define the research, education and advocacy initiatives that EUFOREA, a non—profit organization dedicated to improving respiratory care, will develop over the next 2 years leading up to its 10th anniversary in 2025. In line with the mission to improve healthcare, the Expert Panels on Asthma, AR, Allergen Immunotherapy and Paediatrics, CRS, together with the EPOS group (European Position Paper on Rhinosinusitis and Nasal Polyps), have proposed and elaborated a series of activities corresponding to the main unmet needs in the field of allergy and respiratory care. Their report provides a concise overview of EUFOREA's achievements, ambitions and an action plan for the future, enabling all stakeholders in the field of allergy and respiratory diseases to be updated and inspired to join forces in Europe and beyond.

## Conclusion

The articles in this Research Topic make valuable contributions to research and clinical practice in the field of upper and lower airway pathology. By examining the complex interplay of contributing factors, these studies highlight the need for personalized and comprehensive care, as well as the need for research to improve diagnosis, treatment and patient outcomes.

In addressing the challenges proposed by the authors, it is increasingly clear that current trends need to be directed towards patient-centred care. Much remains to be done, but this Research Topic makes its contribution to the field.
